# A pH-stable positively charged composite nanofiltration membrane with excellent rejection performance

**DOI:** 10.1039/c9ra06528h

**Published:** 2019-11-18

**Authors:** Zhibin Jiang, Jing Miao, Yuantao He, Xinjun Hong, Kai Tu, Xi Wang, Shunquan Chen, Hao Yang, Ling Zhang, Rui Zhang

**Affiliations:** Guangdong Key Laboratory of Membrane Materials and Membrane Separation, Guangzhou Institute of Advanced Technology, Chinese Academy of Sciences Guangzhou 511458 China jing.miao@giat.ac.cn sq.chen@giat.ac.cn niureal@gmail.com +86-2022912525 +86-13829708450; Key Laboratory for Green Chemical Process of Ministry of Education, School of Environmental Ecology and Biological Engineering, Wuhan Institute of Technology Wuhan 430205 China; School of Resource and Environment, University of Jinan Jinan 250022 China chm_zhangl@ujn.edu.cn; R & D Center, Sinochem Ningbo River Membrane Technology Corp. Ltd. China; School of Chemistry and Environment, South China Normal University Guangzhou 510631 China

## Abstract

A novel kind of pH-stable positively charged composite nanofiltration (NF) membrane with excellent rejection performance was developed *via* interfacial polymerization on the surface of a polysulfone (PSF) ultrafiltration (UF) membrane, using a mixture of polyethyleneimine (PEI) and piperazine (PIP) as the monomers of the aqueous phase, and cyanuric chloride (CC) as the monomer of the organic phase. The strong electron withdrawing and steric hindrance effects of the chloride group in the molecules of CC could protect the amido bond from the attack of hydrogen ions (H^+^) or hydroxyl ions (OH^−^) under acidic or alkaline conditions, thus the resultant polyamide composite membranes could be stable in acidic or alkali aqueous solution. A more compact PA active layer could be developed *via* mixing PIP into the PEI aqueous solution, where the PIP molecules could fill the pores of the polymer networks. There was no obvious change in the surface morphologies, the chemical structures, and the rejection performances after immersing the resultant polyamine composite NF membranes in the strong acidic solution (pH 1) and the strong alkaline solution (pH 13) for 30 days, respectively. The rejection performances of this kind of polyamine composite NF membranes could be adjusted through adjusting the mass ratio of PEI to PIP in the aqueous phase.

## Abstract 2

A pH-stable positively charged composite nanofiltration (NF) membrane was developed *via* the interfacial polymerization (IP) between polyethyleneimine (PEI), piperazine (PIP), and cyanuric chloride (CC).

## Graphical abstract

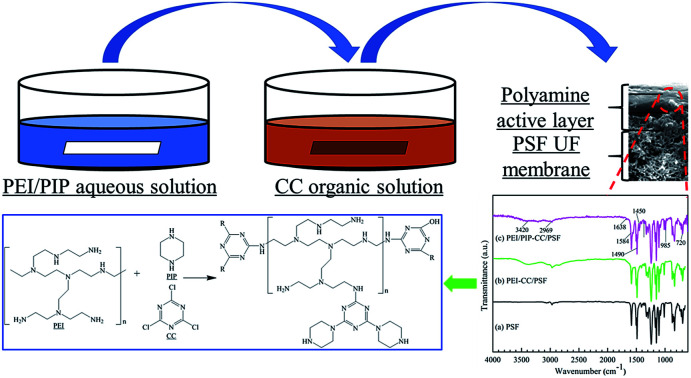

## 1. Introduction

Nanofiltration (NF) has been widely applied to the separation of low-molecular weight organic matter and multi-/divalent ions from monovalent ions, including in wastewater treatment, desalination, water softening and purification, the pharmaceutical and dye industries, *etc.*^1^ Most of the commercial NF membranes are polyamide (PA) and cellulose acetate (CA) composite membranes, fabricated *via* the method of interfacial polymerization on the surface of ultrafiltration (UF) membranes as the supporting substrate for the active layer, or the L–S phase inversion method. Most of the commercial NF membranes are generally used at pH 2–10 or pH 3–9. However, the real industrial operating conditions might be extreme pH conditions, at which the C=O or C–N in the amide bonds would be attacked and destroyed by the H^+^ or OH^−^, and then the amidogen and carboxyl groups are produced. The hydrolyzation is manifested by a severe reduction in rejection performances of the membranes.^2,3^ Zhang, *et al.*^4^ developed a novel organic–inorganic hybrid composite NF membrane based on poly (vinyl alcohol)–aminopropyl triethoxysilane (PVA–APES), and it is the first time that APES was employed in the research of pH-resistant NF membrane. Compared with conventional NF membranes, the PVA–APES hybrid NF membrane exhibited excellent resistance to acidic and alkaline conditions. However, the fabrication process of this kind of hybrid membranes was really complicated, and the compatibility between PVA and APES was unsatisfactory as well. Wu, *et al.*^5^ employed multiwalled carbon nanotube (MWCNTs) and titanium dioxide (TiO_2_) to develop another kind of hybrid composite NF membrane, respectively. It was found that the stability of the resultant hybrid membranes at extreme pH conditions had been improved, but it is also quite difficult to solve the compatibility of organic and inorganic materials as developing this hybrid membranes. Lee, *et al.*^6^ employed cyanuric chloride (CC) as the monomer of the organic phase, instead of trimesoyl chloride (TMC), to develop a pH-stable organic polymer NF membrane, where it was unnecessary to worry about the compatibility between organic and inorganic materials. Based on the previous research, Meng, *et al.*^7^ fabricated a compact polyamidoamine on the surface of the PA selective layer to prevent the attacking from H^+^ or OH^−^.

Therefore, according to the mechanism of PA at acidic or alkaline condition, it could be inferred that electron-donating groups and electron-withdrawing groups could prevent the amide bond from the attacking of H^+^ or OH^−^, respectively. Additionally, the bulky radical groups could suppress such attacking as well. In brief, it is feasible to modify or adapt radical groups around the amide bond to protect it with steric hindrance effect or electrostatic interaction. Actually, the polyamide group is produced by interfacial polymerization between the amine (–NH_2_) group in the aqueous phase and the acyl chloride group (–COCl) in the organic phase. Interestingly, the interfacial polymerization between PEI and CC with the similar molecular structure to TMC could also process quite quickly. Additionally, the PA membranes based on PEI showed higher rejections to positively charged di/multi-valent ions, such as Mg^2+^, Ca^2+^, Cu^2+^, *etc.* The rejection performances of PEI composite NF membranes could be enhanced by doping the amine monomer with low molecular weight in the PEI aqueous phase, such as piperazine (PIP), to obtain a more compact active layer.

In this work, a novel kind of pH-stable positively charged composite NF membrane with excellent rejection performances was developed *via* interfacial polymerization on the surface of a polysulfone (PSF) UF substrate, using the mixture of PEI and PIP as the monomers of the aqueous phase, and CC as the monomer of the organic phase. The rejection performances, the chemical structures, the morphologies, and the stabilities of the resultant PEI composite NF membranes at extreme pH conditions have been investigated systematically and compared with the conventional PA composite NF membranes.

## 2. Experimental section

### 2.1. Materials and chemicals

The PSF UF substrate, with a Molecular Weight Cut-Off (MWCO) of 30 000 Da, were provided by Pureach Tech Ltd (Beijing, China). PEI (*M*_W_: 70 000 Da, 50 wt%), PIP (99.0%), TMC (98.0%), and CC (99.0%) were purchased from Shanghai Macklin Biochemical Co. Ltd., China. *n*-Hexane and all inorganic electrolytes with AR grade, including MgCl_2_, MgSO_4_, NaCl, and Na_2_SO_4_, were purchased from China National Pharmaceutical Group Co., Ltd. (Sinopharm), China. Milli-Q water was used for the preparations and the tests for rejection performances. All chemicals were used without further purification.

### 2.2. Membrane preparation

Part 1: the positively charged PA-based composite NF membranes were fabricated *via* interfacial polymerization (IP).^8^ The preparation process was described as the following. The PSF UF substrates were immersed in 3.7 wt% PEI aqueous solution for 5 min, and then the excess aqueous solution was removed. The membranes were dried at 30 °C for 30 min. After drying, the membranes were immersed for 120 s in 0.25 w/v% TMC/*n*-hexane solution for IP. After IP, the resultant composite NF membrane was rinsed extensively, labeled as PEI-TMC/PSF, and then stored for further uses.

Part 2: the pH-stable positively charged composite NF membranes were fabricated with the similar preparation process described in Part 1. The differences were the monomers in the aqueous phase and the organic phase. The membrane samples prepared using the aqueous phase with the mass ratios of PEI to PIP of 0 : 1, 1 : 1, and 1 : 0, were labeled as PIP-CC/PSF, PEI/PIP-CC/PSF, and PEI-CC/PSF, respectively.

### 2.3. Characterizations of the resultant membranes

The surface and the cross-section morphologies of PSF UF substrate and the positively charged PEI-TMC/PSF, PIP-CC/PSF, PEI/PIP-CC/PSF, and PEI-CC/PSF composite NF membranes, were observed with scanning electron microscope (SEM, Phenom XL, Netherlands). Before observation, the membrane samples were fractured in liquid nitrogen, and then sprayed with gold on the surface using a JS-16009 ion sputter. The 3-D morphology and the roughness of the membrane surface were observed and measured on an atomic force microscope (AFM, SPM-9700, Shimadzu Corp., Japan). The chemical structures of the membranes were investigated with an attenuated total reflectance-Fourier transform infrared (ATR-FTIR) spectroscope (Nicolet iS10, Thermo Fisher Scientific, the United States). The hydrophilicities of the membranes were characterized with a water contact angle (CA) goniometer (DSA30, KRÜSS, Germany). The electro-kinetic characteristic of the membrane surface was characterized with an electrokinetic analyzer (SurPASS™ 3, Anton Paar GmbH, Austria) at the pH in the range of 2 to 10, using 0.001 mol L^−1^ KCl aqueous solution. The surface zeta potential was calculated according to the Helmholtz–Smoluchowski equation with the Fairbrother and Mastin substitution.^9^

### 2.4. Rejection performances of the resultant composite NF membranes

The rejection performances of the resultant composite NF membranes were evaluated with a cross-flow filtration equipment with an effective membrane area of 70 cm^2^. The concentration of inorganic electrolyte and the PEG with different molecular weights (MWs) herein are 1 g L^−1^. The rejection (*R*) and the permeation flux (*F*) were calculated with the following equations.1
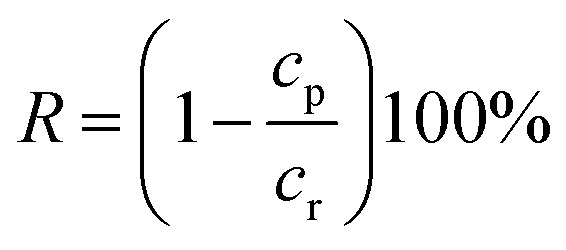
2
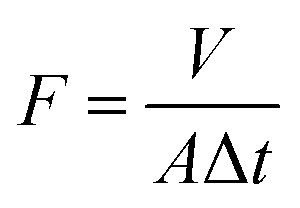
where, *F* is the permeate flux (L m^−2^ h^−1^), *V* is the volume of permeation fluid (L), Δ*t* is the permeation time (h), *A* is the effective permeation area (m^2^), *R* is the rejection (%), *c*_p_ and *c*_r_ are the concentrations of the permeation and the feed solution (g L^−1^), respectively. The conductivity was determined with an electrical conductivity meter (DDS-307, Inesa, China) to get the concentration of inorganic electrolyte *via* the standard curve of electrical conductivity *vs.* the concentration of inorganic electrolyte aqueous solution.

### 2.5. MWCO measurement

Herein, the PEG aqueous solutions of MWs in the range of 400 to 2000 Da were employed to determine the MWCO of the PEI/PIP-CC/PSF composite NF membrane. The concentrations of the PEG with different MWs were measured with the ultraviolet-visible spectro-photometer (UV-6100, MAPADA, China). The membrane pore size (*r*_p_) could be obtained from Eqn (3).^10^

3 log *r*_s_ = −1.3363 + 0.295 log *M*_w_

### 2.6. Acid and alkali-resistance performances of the resultant composite membranes

The PEI-TMC/PSF and PEI-CC/PSF composite NF membranes were immersed in 300 mL 0.1 M HNO_3_ (pH 1) and NaOH (pH 13) aqueous solutions for 30 days at ambient temperature. After the immersion, the membrane samples were washed and rinsed with water. Then the morphologies, chemical structures, and the rejection performance of the membrane samples were characterized and evaluated, respectively. The rejection performances were evaluated at 1.0 MPa and ambient temperature, using 1 g L^−1^ NaCl, MgSO_4_, MgCl_2_, and Na_2_SO_4_ as the inorganic electrolytes. The results were compared with those of PA composite NF membranes.

## 3. Results and discussions

### 3.1. Characterizations of membranes

#### 3.1.1. Membrane morphologies

**Fig. 1:**
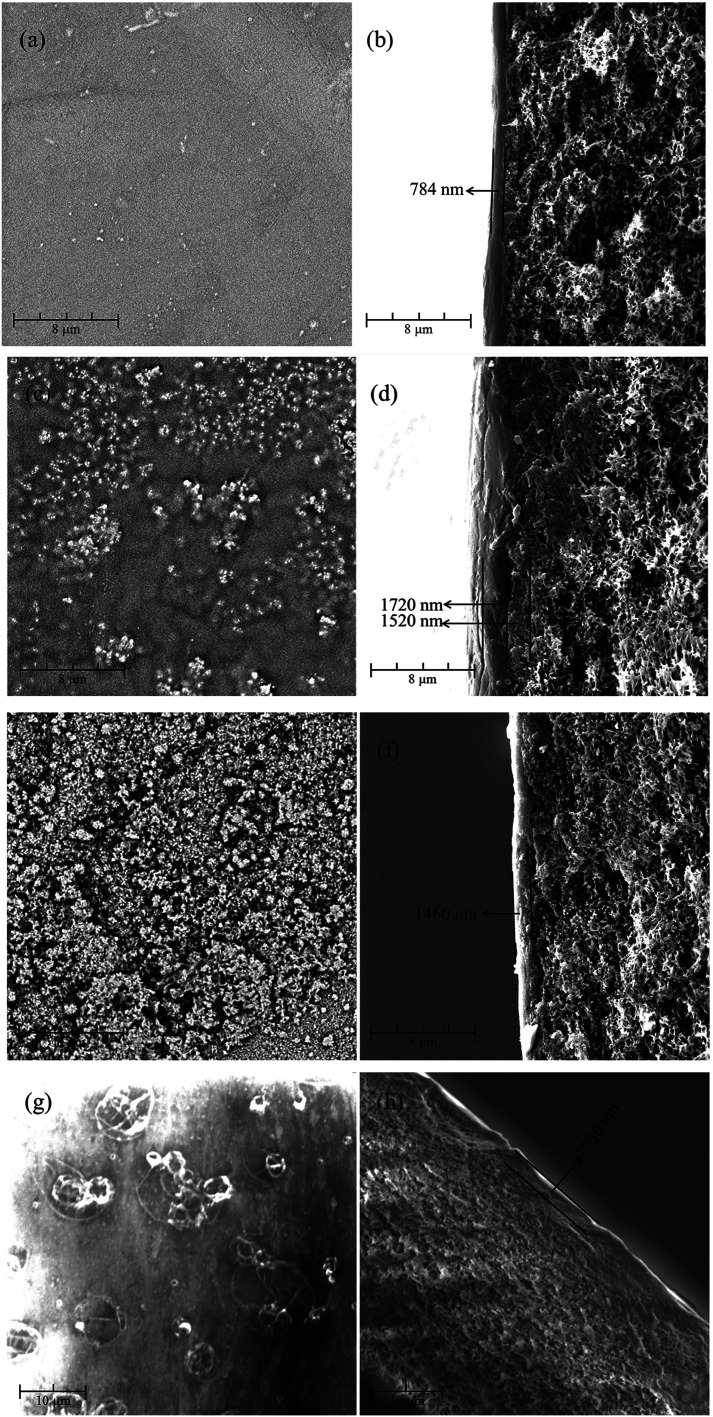
The surface and the cross-section morphologies of (a and b) PEI-CC/PSF, (c and d) PEI/PIP-CC/PSF (e and f) PIP-CC/PSF, and (g and h) PEI-TMC/PSF composite NF membranes.

SEM was employed to observe the surface and the cross-section morphologies of the resultant positively charged composite NF membranes, including PEI-CC/PSF, PEI/PIP-CC/PSF, PIP-CC/PSF, and PEI-TMC/PSF. Fig. 1(a–f) show the results. The roughness of the membranes' surfaces increased with the increase of the mass ratio of PIP to PEI. The active layer thicknesses of PIP-CC/PSF, PEI/PIP-CC/PSF, and PEI-CC/PSF composite NF membranes were 3240 nm, 1460 nm, and 784 nm, respectively. Especially, the PEI/PIP-CC/PSF composite NF membrane was constituted by two different active layers. The inward one was similar to the PIP-CC, and the other might be the PEI-CC layer. The images showed in Fig. 1(g–h) were the surface and cross-section morphologies of the PEI-TMC/PSF composite NF membrane. It could be seen that there are some agglomerates on the membrane surface of PEI-TMC/PSF, and the active layer thickness was approximate 1730 nm.^8,10–14^

According to the results above, it could be inferred that the PIP molecules might diffuse into the hole in PSF UF substrate due to its low molecular weight, and react with CC to form the inward active layer, while the PEI molecules stayed on the substrate surface to react with CC to form the outward active layer. Therefore, the active layer of the PEI/PIP-CC/PSF composite NF membrane was composed of two different polymers.^15–17^

#### 3.1.2. 3-D morphology and surface roughness of the resultant NF membrane

**Fig. 2:**
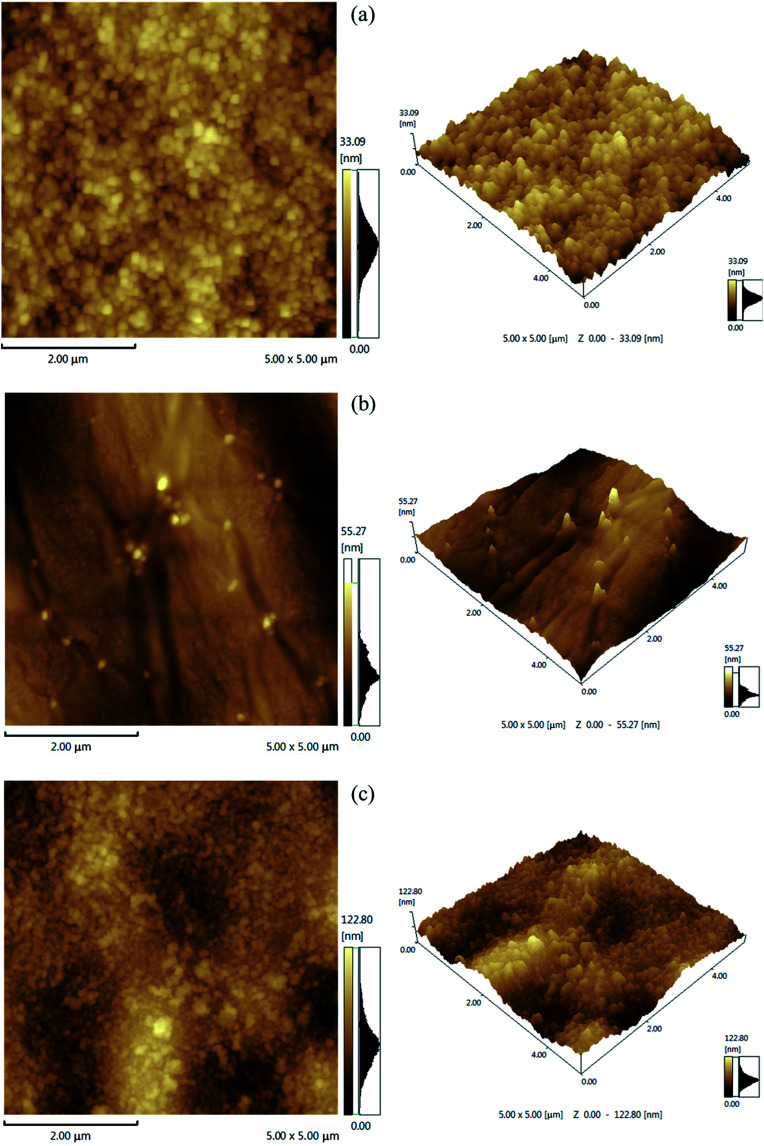
AFM images (5 μm × 5 μm) of (a) PEI-CC/PSF, (b) PEI/PIP-CC/PSF, and (c) PIP-CC/PSF composite NF membranes.

The 2-D and 3-D surface morphologies of the positively charged PEI-CC/PSF, PEI/PIP-CC/PSF, and PIP-CC/PSF composite NF membranes were scanned at three different locations and three times at each location with AFM. The analysis of 2-D and 3-D surface morphologies were in accordance with the SEM results. The average values of the root mean square roughness (*R*_rms_) were 3.16 nm, 5.51 nm, and 12.39 nm, respectively, and the surface roughness increased with the increase of the PIP mass ratio in the aqueous phase. Generally, the higher the roughness of the membrane surface is, the higher the permeate flux is (Fig. 2).^11,13^

#### 3.1.3 ATR-FTIR spectra

**Fig. 3:**
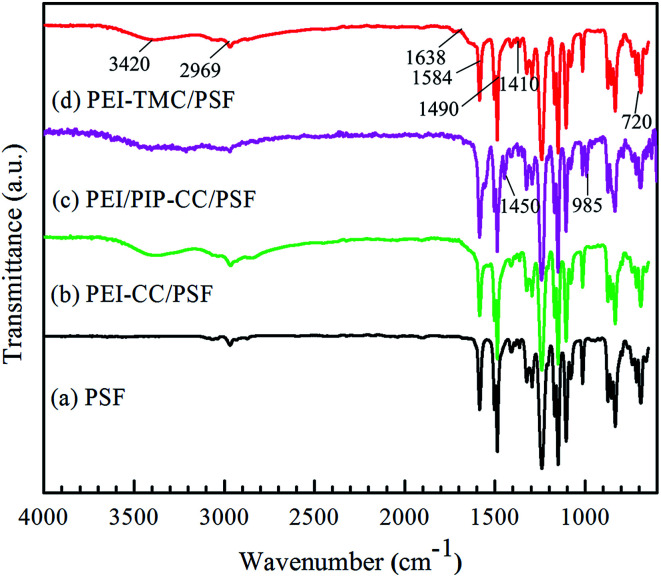
ATR-FTIR spectra of (a) PSF UF substrate, (b) PEI-CC/PSF, (c) PEI/PIP-CC/PSF, and (d) PEI-TMC/PSF composite NF membranes.

The ATR-FTIR spectra of PSF UF substrate, PEI-CC/PSF, PEI/PIP-CC/PSF, and PEI-TMC/PSF composite NF membranes were shown in Fig. 3. Comparing the spectrum of PSF UF substrate with those of the resultant composite NF membranes, a new strong absorbance peak appeared at 3420 cm^−1^, which could be attributed to the N–H stretching of amine groups, and the O–H stretching of hydroxyl groups.^18^ The absorbance peak appeared at 1686 cm^−1^ could be attributed to the C=O stretching of amide bond, but it could not be observed in Fig. 3(b) and (c).^16,17,19,20^ The distinctive IR bands at 1584 cm^−1^, 1410 cm^−1^, and 720 cm^−1^ could be assigned to the ring stretch and the deformation vibrations arising from the triazine ring.^6^ Comparing Fig. 3(b) with Fig. 3(c), there were two new obvious absorbance peaks appeared at 1450 cm^−1^ and 958 cm^−1^, being attributed to the C–H and N–H stretching of PIP molecules. Additionally, it could be found that the absorbance peaks at 1458 cm^−1^ in Fig. 3(b)–(d) were wider than that in Fig. 3(a), which could be attributed to the characteristic peak of the C–N stretching. Hence, it could be concluded that the interfacial polymerization have happened between PEI/PIP and CC, and the polyamine active layers have been formed on the surface of the PSF UF substrate (Table 1).^21^

**Table 1:** Root mean square roughness (*R*_rms_) of the resultant composite NF membranes

Membranes	*R*_rms_ (nm)
PEI-CC/PSF	3.16 ± 0.06
PEI/PIP-CC/PSF	5.51 ± 0.03
PIP-CC/PSF	12.39 ± 0.09

#### 3.1.4. Hydrophilicites of the membrane surfaces

As seen from Table 2, the membranes developed using CC as the monomer of the organic phase was more hydrophobic than those prepared using TMC. It is well known that the membranes' surface wettability is depended on the amount of hydrophilic groups in the active layer surface and the roughness of membrane surfaces. The amount of the hydrophilic groups on the surface of the PIP-CC/PSF composite NF membrane was less than those of PEI-CC/PSF and PEI/PIP-CC/PSF composite NF membrane. However, it could be known from the results of the contact angles that the hydrophilicities of these membrane surfaces increased with increasing the mass ratio of PIP in the aqueous phase. Thus the hydrophility of the PEI/PIP-CC/PSF composite NF membranes were hinged by the roughness of membrane surface.^22–25^

**Table 2:** Contact angle of different membranes

Membranes	Contact angle (°)
PSF	75.1 ± 0.6
PEI-CC/PSF	76.3 ± 0.3
PEI/PIP-CC/PSF	64.2 ± 0.6
PIP-CC/PSF	58.8 ± 0.1

#### 3.1.5. Electrokinetic properties of the membranes surfaces

**Fig. 4:**
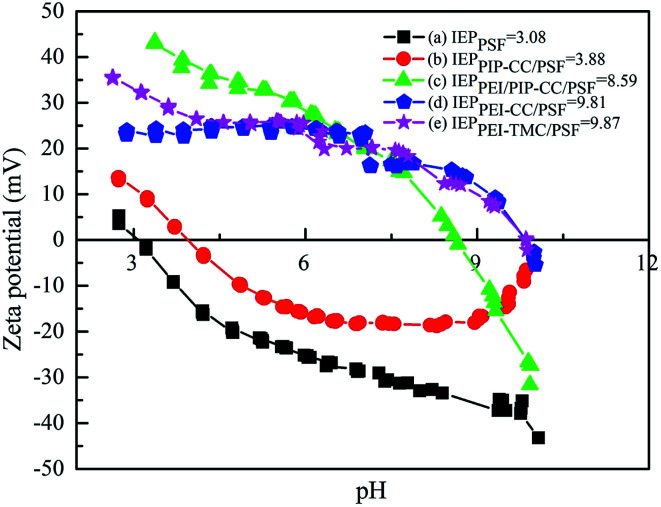
Surface zeta potentials of (a) PSF UF substrate, (b) PIP-CC/PSF, (c) PEI/PIP-CC/PSF, (d) PEI-CC/PSF, and (e) PEI-TMC/PSF membranes.

It could be known from Fig. 4 that the isoelectric points (IEP) of the PSF, PIP-CC/PSF, PEI/PIP-CC/PSF, PEI/PIP-CC/PSF, and PEI-TMC/PSF composite NF membranes were 3.08, 3.88, 8.59, 9.81, and 9.87 mV, respectively.^26,27^ The IEP values of these resultant positively charged composite NF membranes decreased with the increase of PIP mass ratio in the aqueous phase.^15^ The IEP results suggested that the charge properties of the resultant NF membranes would not be changed after replacing TMC with CC. The composite NF membranes prepared *via* the interfacial polymerization between PEI or PEI/PIP and CC are still positively charged, and the PEI/PIP-CC/PSF composite NF membrane showing higher rejections to divalent-valence and multi-valence positive ions.^21^

#### 3.1.6. MWCO of the resultant PEI/PIP-CC/PSF composite NF membrane

**Fig. 5:**
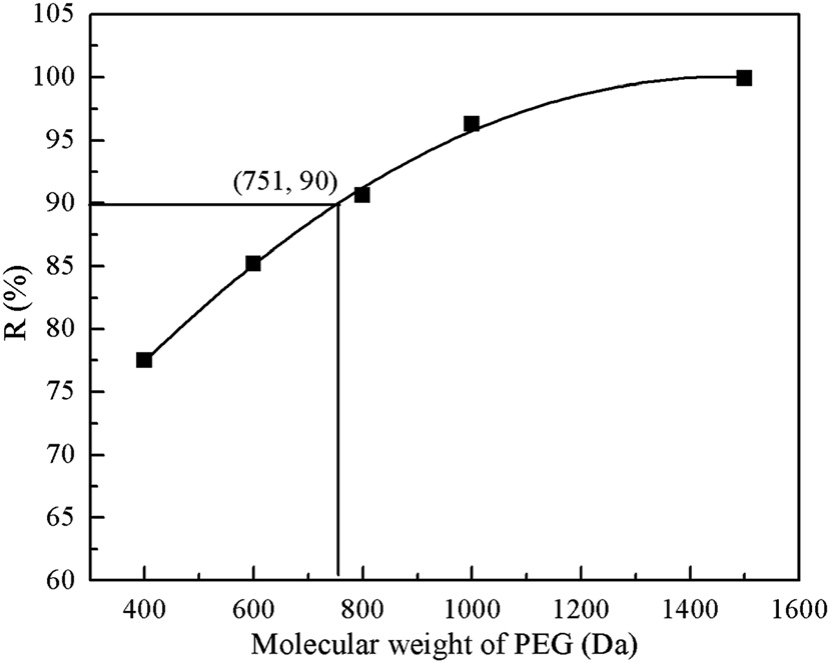
The rejections of the resultant composite NF membrane to PEG of different MWs.

Fig. 5 showed the rejections of the resultant composite NF membrane to PEG with different MWs. It could be seen that the MWCO of PEI/PIP-CC/PSF composite NF membranes was 751 Da, corresponding to the effective membrane pore size (*r*_p_) of 0.63 nm.

### 3.2. Rejection performances of the resultant composite NF membranes

**Fig. 6:**
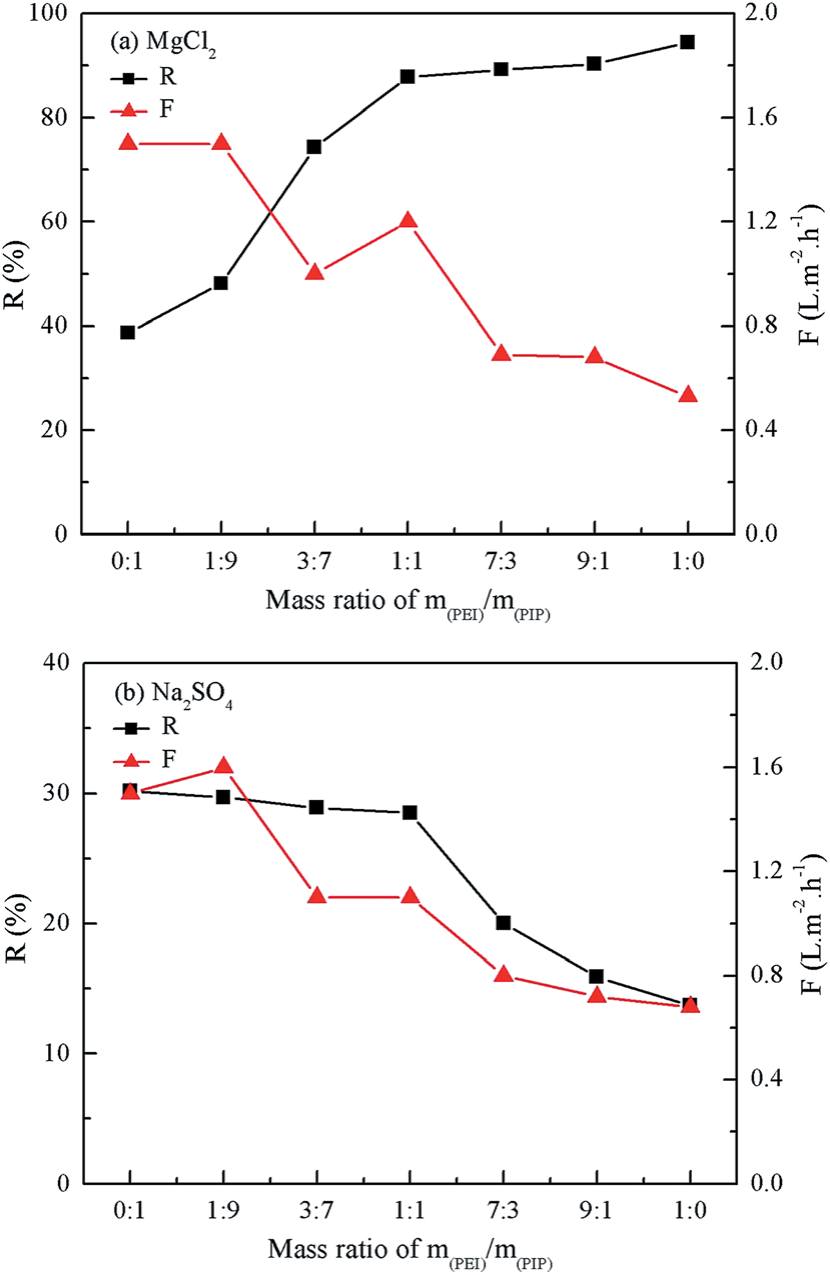
Effect of the mass ratio of PEI/PIP on the rejection of the resultant composite NF membranes to MgCl_2_ and Na_2_SO_4_ aqueous solution.

As seen from Fig. 6, the rejection to 1 g L^−1^ MgCl_2_ aqueous solution decreased with the decrease in the mass ratio of PEI, whereas the rejection to 1 g L^−1^ Na_2_SO_4_ aqueous solution increased. The addition of PIP in the aqueous phase is beneficial to the compactness of the active layer, thus the rejection to Na_2_SO_4_ increased as well. The mass ratio of PEI to PIP being 1 : 1, the rejection to 1 g L^−1^ Na_2_SO_4_ aqueous solution was increased to 28.5% from 13.7%. However, the rejection to Na_2_SO_4_ did not increased any more as increasing the mass ratio of PIP further, while the rejection to MgCl_2_ decreased from 87.8% to 74.3%. The rejections of the positively charged PEI/PIP-CC/PSF composite NF membrane to inorganic electrolytes followed the order of MgCl_2_ > MgSO_4_ > NaCl > Na_2_SO_4_, due to the Donnan effect and the steric-hindrance effect.^28–30^ The 1 : 1 mass ratio of PEI and PIP was selected as the optimal to fabricate the membrane with the acid and alkali-resistance properties and high rejection to inorganic electrolytes.

### 3.3. Acid and alkali-resistance performances of the membranes

#### 3.3.1. Membrane morphologies

**Fig. 7:**
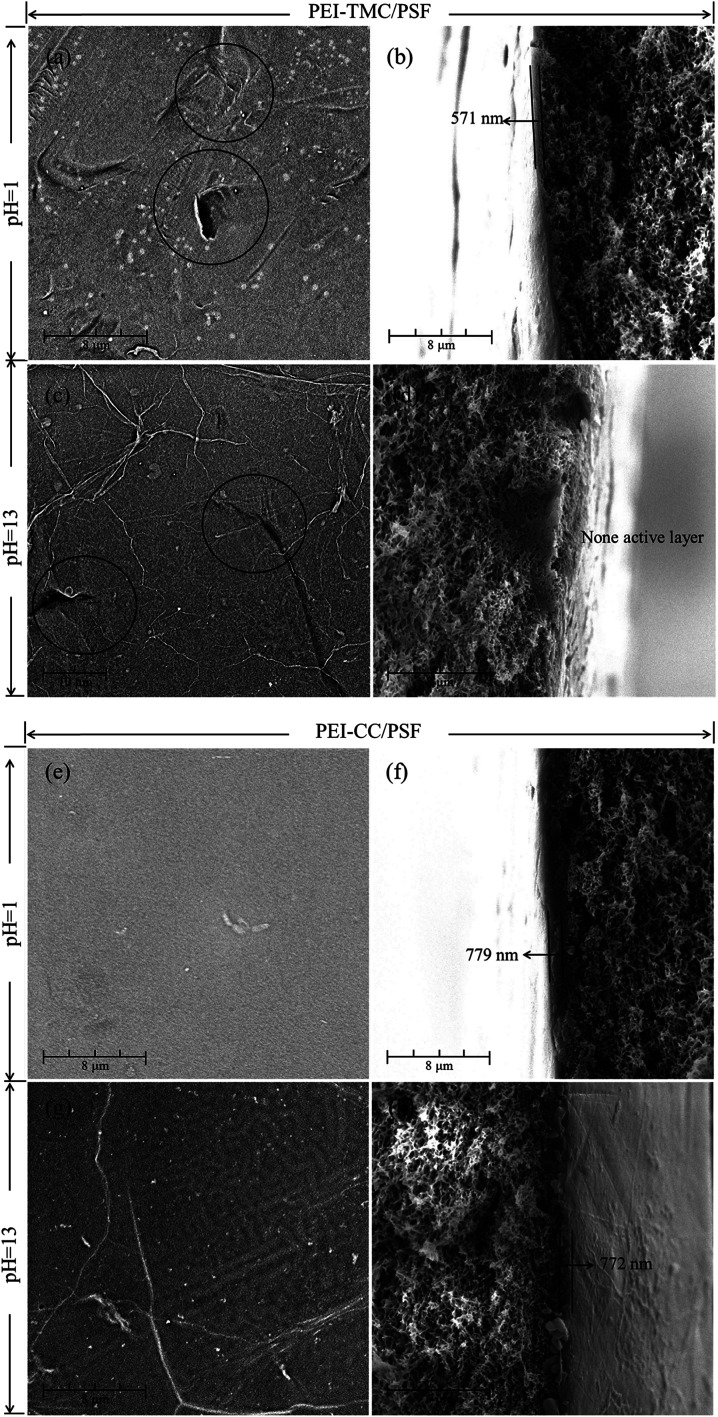
SEM images of the surface and the cross-section for (a–d) PEI-TMC/PSF and (e–h) PEI-CC/PSF composite NF membranes immersed in the solutions with pH 1 and pH 13 for 30 days.

As seen from Fig. 1, there were a lot of agglomerates on the surface of the PEI-TMC/PSF composite NF membrane. Fig. 7(a–d) show the surface and the cross-section morphologies of the PEI-TMC/PSF composite NF membrane after being immersed in the solutions with pH 1 and pH 13 for 30 days. As seen from Fig. 7(a–d), the surface of the PEI-TMC/PSF composite NF membranes, especially the membrane immersed in pH 13 solution, had been seriously corroded. As for the membrane immersed in the aqueous solution with pH 13, the PA active layer could hardly be found. The active layer thickness of the membrane immersed in pH 1 solution was approximate 571 nm, which was much thinner than that of the pristine membrane, 1730 nm.

Compared with the pristine PEI-TMC/PSF composite NF membrane, the active layer of the pristine PEI-CC/PSF composite NF membrane was smoother and thinner. Fig. 7(e–h) show the surface and the cross-section morphologies of PEI-CC/PSF composite NF membranes that had been immersed in pH 1 and pH 13 solution for 30 days. Interestingly, there was not any obvious change in the membranes' surface and the thickness of the active layer. The membranes' thickness just decreased from 784 nm to 779 nm and 772 nm, respectively. It could be concluded that the acidic and alkaline solution are not able to destroy the PEI-CC/PSF composite NF membranes.

#### 3.3.2. Chemical structures of the membranes

**Fig. 8:**
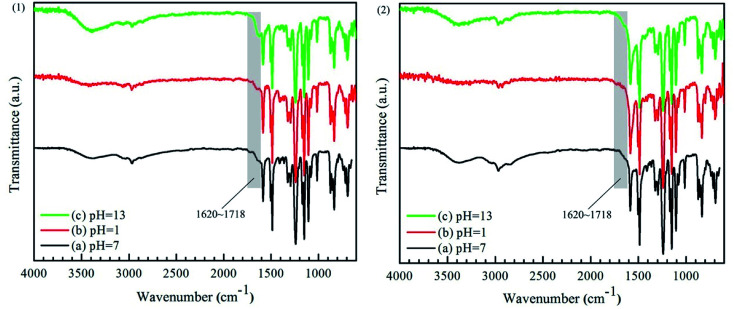
ATR-FTIR spectra of (1) PEI-TMC/PSF and (2) PEI-CC/PSF composite NF membranes immersed in the solutions with pH 1 and pH 13 for 30 days.

As seen from Fig. 8 (1) and (2), the absorption peak in the range of 1620 to 1718 cm^−1^, attributed to the C=O bond in –COOH,^15^ had been strengthen. However, there was not any change observed in the ATR-FTIR spectra of PEI-CC/PSF composite NF membrane before and after the immersions in the aqueous solutions with different pH values for 30 days. It could be concluded that the chemical structure of PEI-CC/PSF composite NF membrane is not destroyed under both acidic and alkaline conditions, while PA active layer in PEI-TMC/PSF composite NF membrane would hydrolyze to produce –NH_2_ and –COOH at similar conditions.^26,27,31,32^

#### 3.3.3. Rejection performances

As known from Table 3, the rejections of PEI-TMC/PSF and PEI-CC/PSF composite NF membranes decreased after the immersions in pH 1 and pH 13 aqueous solutions for 30 days, respectively. However, the decrease of PEI-CC/PSF composite NF membrane was much lower than that PEI-TMC/PSF. Therefore, the PEI-CC/PSF composite NF membrane was much more stable than that of PEI-TMC at the extremely pH conditions.

**Table 3:** Rejections and permeation flux to inorganic electrolytes after immersing the membranes in the solutions with different pH values for 30 days

Electrolytes	pH values	Membrane samples			
		PEI-CC/PSF		PEI-TMC/PSF	
		*R* (%)	*F* (L m^−2^ h^−1^)	*R* (%)	*F* (L m^−2^ h^−1^)
MgCl_2_	1	90.5	0.52	70.3	1.21
	7	94.4	0.53	95.1	1.02
	13	89.6	0.51	54.8	1.50
MgSO_4_	1	87.6	0.50	66.9	1.28
	7	90.5	0.51	92.8	1.01
	13	88.7	0.52	49.3	1.62
NaCl	1	42.8	0.52	30.2	1.33
	7	43	0.53	48.8	1.18
	13	39.6	0.53	27.6	1.72
Na_2_SO_4_	1	27.6	0.51	16.1	1.34
	7	28.7	0.50	33	1.06
	13	26.3	0.50	10.5	1.73

**Fig. 9:**
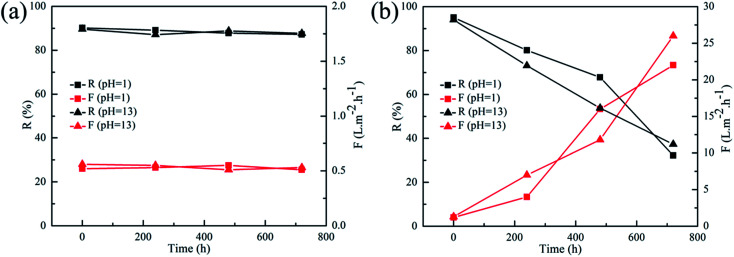
Long term pH stability tests on (a) PEI-CC/PSF and (b) PEI-TMC/PSF composite membranes after prolonged operation in HNO_3_ (pH 1) and NaOH solutions (pH 13) (1 g L^−1^ MgCl_2_, at 1.0 MPa and ambient temperature).

To evaluate the membranes' pH-stable property, the rejection performances of PEI-TMC/PSF and PEI-CC/PSF composite NF membranes were evaluate with HNO_3_ (pH 1) and NaOH (pH 13) aqueous solutions at 1.0 MPa, which contained 1 g L^−1^ MgCl_2_ for 30 days. It could be seen from Fig. 9 that the rejection performances of the PEI-CC/PSF composite NF membranes were quite stable during the whole operation period, while the rejection (*R*) of PEI-TMC/PSF composite NF membrane decreased sharply, and the permeate flux (*F*) increased significantly. It might be because that the PA active layer could be destroyed more easily during the long-term operation, which is quite similar to the work reported by Lee, *et al.*^17^

## 4. Conclusions

In this work, a novel kind of pH-stable positively charged composite NF membranes with excellent rejection performances were successfully developed *via* interfacial polymerization on the surface of the PSF UF membrane, using a mixture of PEI and PIP as the monomers of the aqueous phase, and CC as the monomer of the organic phase. There was no obvious change in the morphologies, the chemical structures, and the rejection performances of the PEI-CC/PSF composite NF membrane after immersing the membrane in the aqueous solutions with extremely pH values. As the PIP molecule was introduced into the membranes' active layer by mixing it with PEI in the aqueous phase, the rejection to 1 g L^−1^ Na_2_SO_4_ aqueous solution increased approximate 14.8%.

## Conflicts of interest

There are no conflicts of interest to declare.

## Abbreviation

AFM: Atomic force microscopy
ATR-FTIR spectroscopy: Attenuated total reflectance-Fourier transform infrared spectroscopy
CA: Contact angle
IP: Interfacial polymerization
NF: Nanofiltration
*F*
: Permeate flux
PSF: Polysulfone
PEI: Polyethyleneimine
PA: Polyamide
*R*
: Rejection
*R*_rms_
: Root mean square roughness
SEM: Scanning electron microscopy
CC: Cyanuric chloride
UF: Ultrafiltration

## Supplementary Material
